# The effects of team-based learning on nursing students’ learning performance with a focus on high-risk pregnancy in Korea: a quasi-experimental study

**DOI:** 10.4069/kjwhn.2021.11.16

**Published:** 2021-12-15

**Authors:** Sunhee Lee, Hyun Jung Park

**Affiliations:** 1 Department of Nursing, Gimcheon University, Gimcheon, Korea; 2 Department of Nursing, Pyeongtaek University, Pyeongtaek, Korea

**Keywords:** Communication, High-risk pregnancy, Learning, Nursing students, Problem solving

## Abstract

**Purpose:**

The purpose of this study was to examine the effects of team-based learning (TBL) on nursing students’ communication ability, problem-solving ability, self-directed learning, and nursing knowledge related to high-risk pregnancy nursing.

**Methods:**

This quasi-experimental study used a nonequivalent control group pretest-posttest design. The participants were 91 nursing students allocated to an experimental group (n=45) and a control group (n=46). The experimental group received TBL lectures three times over the course of 3 weeks (100 minutes weekly) and the control group received instructor-centered lectures three times over the course of 3 weeks (100 minutes weekly). Data were collected by questionnaires from September 10 to November 8, 2019. Data were analyzed using the chi-square test, paired t-test, and independent t-test.

**Results:**

After the intervention, the mean scores of problem-solving ability (t=–2.59, *p*=.011), self-directed learning (t=4.30, *p*<.001), and nursing knowledge (t=3.18, *p*=.002) were significantly higher in the experimental group than in the control group. No significant difference in communication ability was found between the experimental and control group (t=1.38, *p*=.171)

**Conclusion:**

The TBL program was effective for improving nursing students’ problem-solving ability, self-directed learning, and nursing knowledge. Thus, TBL can be considered an effective teaching and learning method that can improve the learning outcomes of high-risk pregnancy nursing in women’s health nursing classes. The findings suggest that TBL will be helpful for future nursing students to develop the nursing expertise necessary for providing nursing care to high-risk pregnant women.

## Introduction

In response to recent increases in acute, chronic, and infectious diseases, as well as rapid changes in the medical environment, nurses must be able to provide integrated nursing care in the field of clinical nursing. Therefore, international institutions of higher medical education are actively trying to enhance the necessary capabilities of medical professionals by applying learner-centered educational methods, such as problem-centered learning and team-based learning (TBL), which focus on interactions between small groups [1].

Moreover, universities in Korea are expanding learner-centered instructional methods, such as problem-centered learning, TBL, and action learning, to improve university students’ competency [2]. As a result of these trends in Korea and abroad, to enhance the core competencies of nurses, there is an increasing necessity for learner-based classes to be developed and applied in the field of nursing in Korea.

Life skills, which are a core competency that one develops throughout life, include communication ability, problem-solving ability, self-directed learning, and leadership [3]. TBL is a learner-centered and self-directed active educational strategy, which is expected to equip nursing students with the ability to apply their experience in rapidly changing situations in clinical nursing by improving their understanding of complicated clinical emergency situations, knowledge, problem-solving ability, communication ability, academic achievement, and class satisfaction [4].

In recent years, TBL has also been widely used internationally [5-7]. According to Branney and Priego-Hernández [5], in a pathophysiology class that combined the use of TBL and the traditional learning method for 197 nursing students, higher responsibility and satisfaction were observed with TBL. They argued that TBL was an effective educational method that can encourage students to engage in active learning. Alberti et al. [6] reported in their systematic review of 12 studies applying TBL in nursing that 10 studies had a quasi-experimental design, nine studies showed improvements in academic achievement and nursing skills, and seven studies described improvements in communication ability, learning ability for the professional field, and self-directed learning ability. Dearnley et al. [7] stated in their systematic review of 16 studies that applied TBL in midwifery courses that TBL increased students’ participation and satisfaction of the students, led to the development of practical training and changes in the educational method, and that a consistent and structured approach is necessary its application.

In previous studies, TBL in Korean nursing education was mainly applied in simulation training classes [8,9] and various fields of nursing, including nursing major courses and theoretical classes on basic nursing science subjects. Positive effects have been reported for variables such as problem-solving ability, critical thinking, academic achievement, communication ability, and self-directed learning [10,11].

Although the scope of TBL application is being expanded in nursing education due to its educational effects, and TBL has been conducted in women’s health nursing classes focusing on nursing care for normal pregnant women and fetal assessments [12,13], there have not been enough studies applying TBL to nursing for high-risk pregnancies, other than the study by Kim [14]. Due to the increase of delayed marriages and advanced-maternal-age pregnancies, the rate of high-risk pregnancies is steadily rising in Korea [15]. Since high-risk pregnancies lead to complications and are associated with high-risk births, the maternal mortality rate is therefore also increasing [16].

To keep pace with changes in the medical environment and health problems, it is necessary for nurses to go beyond providing maternity nursing focused on normal deliveries; instead, nurses need to distinguish between normal and abnormal pregnancies and provide intensive nursing care to pregnant women in emergency situations or in need of continuous care [17]. Thus, this study aimed to apply TBL in a women’s health nursing class where students learned about diseases associated with high-risk pregnancies, identify its effects on communication ability, self-directed learning ability, problem-solving ability, and nursing knowledge, and help university nursing students enhance their professional competencies needed for high-risk pregnancy nursing to contribute to improving women’s health.

This study aimed to identify the effects of TBL in a women’s health nursing class on high-risk pregnancy nursing, with a specific focus on its effects on students’ communication ability, problem-solving ability, self-directed learning ability, and nursing knowledge.

This study had the following hypotheses:

•Hypothesis 1: The experimental group, in which TBL was applied, would have a higher communication ability score than the control group that received instructor-centered lectures.

•Hypothesis 2: The experimental group would have a higher problem-solving ability score than the control group.

•Hypothesis 3: The experimental group would have a higher self-directed learning ability score than the control group.

•Hypothesis 4: The experimental group would have a higher nursing knowledge score than the control group.

## Methods

### Ethics statement

Ethics statement: This study was approved by the Institutional Review Board of Gimcheon University (No. GU-201908-HRa-10-02-P). Informed consent was obtained from the participants

### Study design

**Figure 1.**
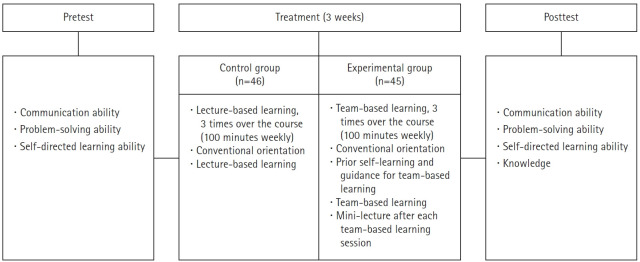
Research design.

This study used a quasi-experimental nonequivalent control group pretest-posttest design (Figure 1). This study report followed the TREND (Transparent Reporting of Evaluations with Nonrandomized Designs) reporting guidelines [18].

### Participants

The participants were junior-year nursing students taking a women’s health nursing class at Gimcheon University, Korea, who consented in writing to participate in the study. Students who enrolled in the women’s health nursing class but did not consent to participate in the study and those who were retaking the women’s health nursing class were excluded. For voluntary participation, the researcher explained the objectives and methods of the study on an “extracurricular day” at the university outside of class hours, and a research assistant collected the written consent forms and post-surveys of the students, which were submitted in a box. The students received an explanation that the surveys on nursing knowledge and other topics, which were conducted at the time of TBL in the experimental group, were not related to their grades and their responses would not result in any disadvantages (including in terms of grades) and that participants could withdraw from the study at any time without any disadvantages. Regardless of their participation in the study, all students in both the experimental and control groups took the same classes from each professor and were given grades from each instructor. Participants in this study were provided with predetermined rewards after they finished all the surveys, and the control group was provided with the materials for TBL afterward.

### Sample size

The one-tailed independent-sample t-test was conducted using G*Power version 3.1.9.2 program to estimate the number of participants needed for the study. Using a confidence level (α) of .05, a test power (1-β) of .80, and an effect size (d) of .55 [19], which is a normal level for the independent-sample t-test, a total of 84 participants (42 each for the experiment group and control group) was calculated. Although there were initially 49 participants each in the experimental and control groups, questionnaires were collected from 45 participants in the experimental group and 46 participants in the control group in the end, satisfying the calculated sample size, and the collection rate of questionnaires was 93%.

### Intervention

The experimental and control groups were selected based on the original composition of the classes. Among the four classes allocated at the beginning of the semester, two classes were selected as the experimental group, where the investigator applied TBL, and two classes were selected as the control group, where instructors-centered lectures were given by other instructors. The instructors of the experimental group and control group established a 15-week lecture plan based on discussions, prepared lectures on topics where TBL was applied according to the learning objectives, and created the same lecture materials for each group in advance. The experimental and control groups were each divided into two classes, and since classes were taught by different instructors at the same time in accordance with the schedule, no information about the lectures was shared between the two groups. TBL was conducted three times (2 class hours consisting of 100 minutes per session, for a total of 6 class hours over the course) on topics such as placenta previa and placental abruption in week 4, gestational diabetes in week 6, and preterm labor and premature rupture of membranes in week 9.

All students, including those in the experimental and control groups, who took the women’s health nursing class received the same lectures from each instructor, and only the participants in the study completed the additional surveys outside of class hours. There was no disadvantage associated with allocation to the experimental or control group since the surveys on nursing knowledge and other outcomes were not reflected in their grades, which were given by the corresponding instructor.

In this study, three topics about high-risk pregnancy and births were selected because it was difficult to identify the effects on learning outcomes based on only one session of TBL. The three topics were selected from the fields of high-risk pregnancy and birth, and a total of 18 problems, consisting of six problems for each topic, were prepared. The content validity index (CVI) was measured by three professors of women’s health nursing. The CVI was calculated by evaluating each item on a 4-point Likert scale, and the average CVI was found to be high (.94). Fifteen of the 18 initial items had a CVI of 1.0 and three items had a CVI of .67, and the three items with a CVI less than .80 were excluded.

For stage I (preparation) of the TBL intervention, PowerPoint (Microsoft Corp., Redmond, WA, USA) materials on the learning objectives and main contents of each topic were distributed through a website the week before the application of TBL to give motivation to study. For stage II (readiness assurance), individual readiness for each topic was assessed for 10 minutes at the beginning of the TBL classes. For this stage, 10 multiple-choice items each were administered for bleeding in late pregnancy, premature rupture of membranes, and preterm labor, while the assessment for gestational diabetes was composed of 15 multiple-choice items. Next, the classes were each divided into six groups of four to five participants, who were then given three cases for group discussions. The assessment of group readiness was conducted in the TBL room, and the instructor went around each group to promote group activities and encourage questioning and participation by all learners to promote their engagement. To assess group readiness, four to six subjective problems were given for each case, and the answers to the problems were prepared through group discussions and then submitted. The assessment was conducted for 40 minutes. In stage III (application), intergroup discussions and class discussions were held on the same cases of two groups through presentations by all six groups, and the content was summed up in a mini-lecture at the end. This process lasted for 50 minutes (Table 1).

### Measurement tools

The instruments used in this study were developed by the Korean Educational Development Institute (KEDI) and used in accordance with Free Use of Public Works under the Korea Open Government License of Ministry of Culture, Sports, and Tourism pursuant to Article 24-2 of the Copyright Act.

#### Communication ability

The instrument to measure communication ability for university students/adults developed by KEDI [20] was used. This instrument consists of 49 items, including five ability factors (interpretation ability, role performance ability, self-presentation ability, goal setting ability, and message conversion ability) and seven subfactors (information gathering, attention, overcoming fixed ideas, creative communication/open communication, self-disclosure, and leading communication). The items are scored on a 5-point Likert scale (very rarely, 1 to very often, 5), and a higher total score (range, 49–245) corresponds to higher communication ability. Cronbach’s α when the instrument was developed was .80 [20], and Cronbach’s α in this study was .88.

#### Problem-solving ability

The instrument to measure problem-solving ability measurement for university students/adults developed by KEDI [20] was used. The instrument consists of a total of 45 items including five ability factors (problem clarification, cause analysis, development of alternatives, planning/taking action, and performance assessment) and nine subfactors (problem recognition, information gathering, analyzing ability, divergent thinking, decision-making, planning ability, taking action and risks, evaluation, and feedback). The items are scored on a 5-point Likert scale (very rarely, 1 to very often, 5), and a higher total score (range, 45–225) indicates higher problem-solving ability. Cronbach’s α was .94 at development [20] and .90 in this study.

#### Self-directed learning ability

Self-directed learning ability was measured using 40 items from the instrument to measure self-directed learning ability for university students/adults developed by KEDI, with the exclusion of five items from the subfactor of diagnosis of desire to learn because they did not have a CVI higher than .80 as assessed by two researchers. The instrument consisted of three ability factors (learning plan, learning action, and learning assessment) and eight subfactors (diagnosis of desire to learn, setting learning objectives, identification of resources for learning, basic self-management ability, selection of learning strategies, continuity of learning actions, attribution of efforts for results, and self-examination). With the items scored on a 5-point Likert scale (very rarely, 1 to very often, 5), a higher score indicated a higher level of self-directed learning ability. Cronbach’s α when the instrument was developed was .93 [20], while Cronbach’s α in this study was .79.

#### Nursing knowledge

To measure nursing knowledge in this study, a total of 15 items (five items for each topic, including two short-answer items, two analysis-focused items, and one problem-solving item) were developed by the researcher for the following topics: placenta previa, placental abruption, gestational diabetes, premature rupture of membranes, and preterm labor. The CVI of each item was measured by professors who had taught women’s health nursing for over 10 years, and all items had a CVI of .80 or higher. Correct answers were given a score of 1, and incorrect answers were given a score of 0. The score range was 0 to 15, and a higher total score corresponded to a higher level of nursing knowledge. Cronbach’s α for the reliability of the instrument in this study was .79.

### Data collection

Data were collected from September 10 to November 8, 2019. Among the four classes allocated ㄴat the beginning of the semester, two classes were selected as the experimental group, where TBL was applied by the investigator, and two classes were selected as the control group, where lecture classes were taught by other instructors. The week before the application of TBL, the researcher explained the objectives and methods of the study to the experimental group during class hours and to the control group on “extracurricular day,” and written consent forms were distributed and collected by the students. The surveys for the experimental group and control group were delivered and collected by the students outside of class hours. Preliminary surveys were conducted in both the experimental and control groups using a self-checklist on the week before applying TBL. After the preliminary surveys were collected from the experimental group, they were given preview materials for TBL. Post-surveys were conducted using a self-checklist in the week after TBL had ended.

### Data analysis

Data were analyzed using IBM SPSS ver. 28.0 for Windows (IBM Corp., Armonk, NY, USA). The general characteristics of the subjects were analyzed in terms of frequency, percentage, average, and standard deviation. The chi-square test and t-test were used to test for homogeneity in the general characteristics and dependent variables between the experimental group and control group. To verify the set hypotheses, the independent t-test was used for the effects in the experimental group compared to the control group regarding communication ability, problem-solving ability, and self-directed learning ability before and after TBL. The paired t-test was used for pre-hoc and post-hoc verification in each group. The independent t-test was used to analyze differences between the experimental and control groups in nursing knowledge after TBL, and the reliability of the measurement instruments was tested with Cronbach’s α.

## Results

### Homogeneity testing between the groups for general characteristics and dependent variables

According to the test of homogeneity between the two groups regarding participants’ general characteristics and the dependent variables before the experiment, there were no statistically significant differences in age (t=–1.45, *p*=.336), gender (t=0.30, *p*=.758), personalities (t=0.46, *p*=.73 significant 4), satisfaction with the major (t=–1.24, *p*=.218), satisfaction with interpersonal relationships (t=.32, *p*=.752), communication ability (t=–0.14, *p*=.889), problem-solving ability (t=–0.91, *p*=.367), self-directed learning ability (t=–65, *p*=.519). Thus, the homogeneity of the two groups was verified (Table 2).

### Verification of the effects of TBL

Regarding hypothesis 1, “the experimental group, in which TBL was applied, would have a higher communication ability score than the control group that received instructor-centered lectures,” the experimental group had a communication ability score of 168.51±15.72 and the control group had a score of 162.41±16.24. Although the experimental group had an average score increase of 12.51 after the intervention, whereas the average increase was 7.11 in the control group. The difference was not statistically significant (t=1.38, *p*=.171), so hypothesis 1 was rejected (Table 3).

As to hypothesis 2, “the experimental group, in which TBL was applied, would have a higher problem-solving ability score than the control group that received instructor-centered lectures,” the experimental group had a problem-solving ability score of 167.29±18.40, and the control group had a score of 158.57±16.49. The experimental group had an average score increase of 19.09 after the intervention, while that of the control group was 7.22. There was a statistically significant difference between the two groups (t=–2.59, *p*=.011), supporting hypothesis 2 (Table 3).

Regarding hypothesis 3, “the experimental group, in which TBL was applied, would have a higher self-directed learning ability score than the control group that received instructor-centered lectures,” the experimental group had a self-directed learning ability score of 142.29±17.84, and the control group had a score of 129.61±16.71. The experimental group showed a score increase of 19.64 on average after the intervention, while the score of the control group increased by 5.15. Since there was a statistically significant difference between the two groups (t=4.30, *p*=.001), hypothesis 3 was supported (Table 3).

As to hypothesis 4, “the experimental group, in which TBL was applied, would have a higher nursing knowledge score than the control group that received instructor-centered lectures,” a post-hoc test was conducted in the experimental group. The experimental group had a total knowledge score of 8.13±2.26, which exceeded that of the control group (6.65±2.18) by 1.18. The difference between the two groups was statistically significant (t=3.18, *p*=.002), and hypothesis 4 was therefore supported (Table 4). Although there was no significant difference in the short-answer items (t=0.91, *p*=.364) in the knowledge assessment, there were significant differences in the analysis-focused items (t=2.28, *p*=.0.25) and the problem-solving items (t=4.27, *p*<.001).

## Discussion

This study investigated the effect of TBL on the communication ability, problem-solving ability, self-directed learning ability, and knowledge of junior-year nursing students in a women’s health nursing class. After TBL was applied for three major diseases in women’s health nursing (placenta previa, gestational diabetes, and preterm labor), with three sessions that each lasted 100 minutes, significant differences were found in the nursing students’ problem-solving ability, self-directed learning ability, and knowledge in the analysis-focused items and problem-solving items. However, no significant difference was observed in communication ability.

The finding that TBL did not have a significant effect on improving communication ability led to the rejection of hypothesis 1. Although the communication ability score of the experimental group increased after they engaged in TBL related to women’s health nursing, no significant difference from the control group was found, unlike the study by Kim [14], where students’ communication ability score increased when TBL was applied in high-risk pregnancy nursing education. Rotthoff et al. [21] stated that the communication between medical professionals can be improved through ongoing training, and according to a study by Park [22], the effect of a communication-training program was greatest with a high intervention frequency of twice a week rather than once a week, 4 to 8 training sessions, and a training period of 5 to 8 weeks. In the study by Kim [14], TBL was applied for 1 hour per week for a training period of 8 weeks. The lack of significant differences in this study may therefore be explained by the fact that there were only three sessions, which each lasted for 100 minutes. Therefore, it is necessary to develop TBL that focuses on the ability to communicate in different situations with an increased training period and frequency in the future.

In addition, TBL was effective in improving the problem-solving ability of the nursing students, which is similar to the results of previous studies [9,14,23]. In this study, TBL was applied to the following three topics: preterm labor and premature rupture of membranes, which are emergency situations associated with neonatal mortality; placenta previa and placental abruption, which are emergency situations that cause obstetric bleeding in late pregnancy; and gestational diabetes, which has been increasing recently [16]. The body of a pregnant woman goes through continuous and dynamic changes and adaptations during pregnancy, and over the course of this process, pregestational diseases worsen or new diseases occur, which lead to complications or high-risk births [24]. Following the principles of TBL, the nursing students in this study became active learners; they tried to find information on solutions for emergency nursing problems, analyzed the relevant information, and used comprehensive thinking skills [25]. Moreover, the program emphasized students’ active participation in solving nursing problems that may occur in nursing field, and it was found that their problem-solving ability improved through this learning experience. This improvement in their problem-solving ability will help them respond appropriately to emergency nursing problems as clinical nurses in the field, including maternity wards and delivery rooms, and support pregnant women in delivering safely.

TBL was effective in improving self-directed learning ability in this study, and this result is similar to that of a previous study in which TBL showed effectiveness in self-directed learning [23].

In this study, the students were required to participate in team activities in which they previewed the risk factors and nursing assessment method for high-risk pregnancy diseases and established nursing processes for primary nursing interventions when problems occurred. According to Jun and Ju [23], students’ self-directed learning ability improves as their sense of responsibility increases through the process of solving problems related to the learning topics. Lee [13] pointed out although male students may not be interested in studying women's health nursing since it focuses on women, through TBL, as learner-to-learner and learner-to-professor interactions occur, and as learners participate actively in the learning process, it can serve as an opportunity for male students to increase their interest in women’s health nursing. In a meta-analysis by Lee and Yang [26] of the effects of classes that applied learner-centered instruction methods such as TBL, it was also found that the effects on class-related knowledge and self-directed learning were the greatest.

Finally, the experimental group showed significant differences in nursing knowledge compared with the control group. This is similar to the results of a study by Ulfa et al. [27], where the nursing knowledge score increased when TBL was applied on the topic of nursing care for postpartum bleeding. In particular, in the present study, significant differences were found for analysis-focused and problem-solving items, rather than short-answer items. This finding suggests that TBL is effective for analysis-focused and problem-solving items which require more critical thinking. As the national nursing licensure examination is shifting from problems asking for simple knowledge toward more analysis-focused and problem-solving items [28], TBL will be helpful in improving students’ academic achievement by increasing their thinking ability for problem-solving. This study is meaningful in that it applied learner-centered TBL for women’s health nursing and verified its efficacy, suggesting that TBL is an effective instructional method that can help improve nursing professionals’ academic achievement and enhance their competencies, such as problem-solving ability and self-directed learning ability, by increasing nursing students’ knowledge of nursing for high-risk patients. In addition, TBL will help university nursing students contribute to improving women’s health by enhancing the professional competencies needed in high-risk pregnancy nursing.

However, a limitation of this study is that it did not control for variables related to the instructional skills of the instructors, since the experimental group and control group were taught by different instructors. To overcome this limitation, instructors who had taught women’s health nursing for a number of years taught the control group, and the two instructors managed the class with thorough discussions of the lecture plans and learning outcomes for each class topic before classes. Moreover, since this study tested the effect of TBL applied to only a single course with a limited duration and frequency (only three times), differences in communication ability could not be confirmed, which constitutes a limitation to the interpretation of the study results. Therefore, it is necessary to develop a TBL program with a higher frequency and longer duration period in the future to verify its effects more conclusively.

In conclusion, TBL was an effective instructional method that can improve the knowledge, problem-solving ability, and self-directed learning ability of university nursing students for high-risk pregnancy nursing. Furthermore, TBL will be helpful for improving nurses’ professional competencies for high-risk pregnancy nursing in clinical situations.

## Figures and Tables

**Table 1.** The team-based learning program for high-risk pregnancies

Topic	Content	Teaching strategy	Time (minute)
Introduction to the program	TBL operation plan for 3 topics and guidance on each learning goal	Motivation for learning	20×3 times
	Three topics	Guidance on learning management	
	-Bleeding in late pregnancy		
	-Premature rupture of membranes and preterm labor		
	-Gestational diabetes		
Stage I. Preparation	Individual prior self-learning from PowerPoint presentations	Facilitating self-directed learning	60×3 times
Stage II. Readiness assurance^†^	Individual readiness evaluation: 10–15 multiple-choice questions	Item development	10×3 times
		-Bleeding in late pregnancy: 10 multiple-choice questions	
		-Premature rupture of membranes and preterm labor: 10 multiple-choice questions	
		-Gestational diabetes: 15 multiple-choice questions	
	Group readiness assessment:	Case-based item development	40×3 times
	6 teams with 3 cases discuss 4–6 open-ended questions each	-Bleeding in late pregnancy: 4 subjective questions	
		-Premature rupture of membranes and preterm labor: 10 multiple-choice questions	
		-Gestational diabetes: 15 multiple-choice questions	
		To promote group activities, the instructor circulates among each group and promotes learning	
		Encourage participation of all learners	
Stage III. Application^†^	Intergroup discussion	Facilitating intergroup discussion	50×3 times
	Mini-lecture	Lecturing	

TBL: Team-based learning

† In-class provision.

**Table 2.** Homogeneity test of general characteristics and dependent variables (N=91)

Variable	Categories	Possible score range	n (%) or M±SD						χ^2^ or t	*p*
			Total		Experimental group (n=45)		Control group (n=46)			
Age (year)			21.89±2.82		22.18±3.53		21.59±1.85		1.45	0.336
Gender	Female		72 (79.1)		35 (76.1.)		36 (80.0)		0.30	0.758
	Male		19 (20.9)		10 (21.7)		9 (20.0)			
Personality	Extroverted		36 (39.6)		17 (37.0)		19 (42.2)		0.46	0.734
	Introverted		55 (60.4)		28 (60.9)		27 (60.0)			
Major satisfaction	Positive		55 (60.4)		26 (56.5)		29 (64.4)		–1.24	0.218
	Negative		7 (7.7)		4 (8.7)		3 (6.7)			
	Moderate		29 (31.9)		15 (32.6)		14 (31.1)			
Satisfaction with interpersonal relationships	Positive		59 (64.8)		30 (65.2)		29 (64.4)		0.32	0.752
	Negative		7 (7.7)		3 (6.5)		4 (8.7)			
	Moderate		25 (27.5)		12 (26.1)		13 (28.9)			
Communication ability		49–245	155.08±15.57		154.84±14.47		155.30±16.73		–0.14	.889
Problem-solving ability		45–225	149.79±16.61		148.20±13.14		151.35±19.43		–0.91	.367
Self-directed learning		40–200	123.56±13.29		122.64±13.23		124.46±13.43		–0.65	.519

**Table 3.** Comparison of communication ability, problem-solving ability, and self-directed learning between the two groups

Variable	Group	Mean±SD			t	*p*
		Pretest	Posttest	Difference		
Communication ability	Exp (n=45)	154.84±14.47	168.51±15.72	12.51±18.09	1.38	.171
	Cont (n=46)	155.30±16.73	162.41±16.24	7.11±19.23		
Problem-solving ability	Exp (n=45)	148.20±13.14	167.29±18.40	19.09±18.14	2.59	.011
	Cont (n=46)	151.35±19.43	158.57±16.49	7.22±25.13		
Self-directed learning	Exp (n=45)	122.64±13.23	142.29±17.84	19.64±13.24	4.30	<.001
	Cont (n=46)	124.46±13.43	129.61±16.17	5.15±18.56		

Cont: Control group; Exp: experimental group.

**Table 4.** Comparison of knowledge according to the assessment type between the two groups

Knowledge assessment type	Possible score range	Group	Posttest, mean±SD	t	*p*
Short-answer	0–6	Exp (n=45)	4.11±1.51	0.91	.364
		Cont (n=46)	3.85±1.23		
Analysis-focused	0–6	Exp (n=45)	2.33±1.15	2.28	.025
		Cont (n=46)	1.78±1.15		
Problem-solving	0–3	Exp (n=45)	2.69±1.22	4.26	<.001
		Cont (n=46)	2.02±1.16		
Total	0–15	Exp (n=45)	8.13±2.26	3.18	.002
		Cont (n=46)	6.65±2.18		

Cont: Control group; Exp: experimental group.
